# Neurophysiological modification of CA1 pyramidal neurons in a transgenic mouse expressing a truncated form of disrupted-in-schizophrenia 1

**DOI:** 10.1111/ejn.12549

**Published:** 2014-04-08

**Authors:** Clair A Booth, Jonathan T Brown, Andrew D Randall

**Affiliations:** 1 School of Physiology and Pharmacology, University of BristolMedical Sciences Building, University Walk, Bristol, BS8 1TD, UK; 2 Institute of Biomedical and Clinical SciencesThe Hatherly Building, Exeter, EX4 4PS, UK

**Keywords:** electrophysiology, excitability, hippocampus, psychiatric disease, susceptibility gene, synaptic plasticity

## Abstract

A t(1;11) balanced chromosomal translocation transects the *Disc1* gene in a large Scottish family and produces genome-wide linkage to schizophrenia and recurrent major depressive disorder. This study describes our *in vitro* investigations into neurophysiological function in hippocampal area CA1 of a transgenic mouse (DISC1_tr_) that expresses a truncated version of DISC1 designed to reproduce aspects of the genetic situation in the Scottish t(1;11) pedigree. We employed both patch-clamp and extracellular recording methods *in vitro* to compare intrinsic properties and synaptic function and plasticity between DISC1_tr_ animals and wild-type littermates. Patch-clamp analysis of CA1 pyramidal neurons (CA1-PNs) revealed no genotype dependence in multiple subthreshold parameters, including resting potential, input resistance, hyperpolarization-activated ‘sag’ and resonance properties. Suprathreshold stimuli revealed no alteration to action potential (AP) waveform, although the initial rate of AP production was higher in DISC1_tr_ mice. No difference was observed in afterhyperpolarizing potentials following trains of 5–25 APs at 50 Hz. Patch-clamp analysis of synaptic responses in the Schaffer collateral commissural (SC) pathway indicated no genotype-dependence of paired pulse facilitation, excitatory postsynaptic potential summation or AMPA/NMDA ratio. Extracellular recordings also revealed an absence of changes to SC synaptic responses and indicated input–output and short-term plasticity were also unaltered in the temporoammonic (TA) input. However, in DISC1_tr_ mice theta burst-induced long-term potentiation was enhanced in the SC pathway but completely lost in the TA pathway. These data demonstrate that expressing a truncated form of DISC1 affects intrinsic properties of CA1-PNs and produces pathway-specific effects on long-term synaptic plasticity.

## Introduction

Since the discovery of a balanced chromosomal translocation (1;11) (q42.1;q14.3) segregating with psychiatric disorders in a large Scottish family (Jacobs *et al*., 1970; St Clair *et al*., 1990; Blackwood *et al*., 2001) and the subsequent identification of the *disrupted-in-schizophrenia 1* (*Disc1*) gene (Millar *et al*., 2000), some genetic association studies in family samples and more diverse populations have provided evidence for *Disc1* as a susceptibility gene for psychiatric illnesses including schizophrenia, bipolar disorder and recurrent major depression (Ekelund *et al*., 2004; Hodgkinson *et al*., 2004; Callicott *et al*., 2005; Sachs *et al*., 2005; Thomson *et al*., 2005; Hennah *et al*., 2009). However, other studies, including recent genome-wide association studies, have not identified *Disc1* as a risk factor for psychiatric disorders (Zhang *et al*., 2005; Chen *et al*., 2007; Kim *et al*., 2008; Purcell *et al*., 2009; Mathieson *et al*., 2012) so although still of considerable interest in psychiatry, the role of *Disc1* in such illnesses remains the subject of some debate (Sullivan, 2013).

The protein encoded by the *Disc1* gene is known as DISC1. Numerous splice variants of this protein are likely to exist, although the full-length *Disc1* gene encodes a protein consisting of 854 amino acids with a molecular mass of around 100 kDa. DISC1 expression is reported to be at its highest levels during development but expression persists into adulthood reducing somewhat as animals age. The protein is found in multiple brain regions in adults, including the hippocampus (Austin *et al*., 2004), the subject of this particular study. Functionally, DISC1 appears to be involved in a multitude of cellular processes, including processes that are critical for both brain development and the activity of the adult central nervous system (CNS) (Brandon & Sawa, 2011).

Motivated by its potential role in psychiatric disease, several DISC1-related genetically modified mice have been generated in recent years. These are reported to display a variety of schizophrenia-like phenotypes, both anatomically and behaviourally (Koike *et al*., 2006; Clapcote *et al*., 2007; Hikida *et al*., 2007; Li *et al*., 2007; Kvajo *et al*., 2008; Pletnikov *et al*., 2008; Shen *et al*., 2008). There are, by contrast, very few detailed studies describing neurophysiological phenotypes. Three reports have employed electrophysiological methods to examine intrinsic and synaptic properties in the mouse model developed by Koike *et al*. (2006). These animals express a truncated form of mouse *Disc1* derived from a spontaneous mouse mutation. Findings include decreased early phase long-term potentiation (LTP) in the Schaffer collateral commissural (SC) pathway of the hippocampus (Kvajo *et al*., 2008) and modified short-term plasticity in the mossy fibre pathway (Kvajo *et al*., 2011), both observed with extracellular recording. Cellular level patch-clamp recordings in this mouse have also indicated decreased excitability of dentate granule cells (Kvajo *et al*., 2011), and alterations in the frequency of spontaneous synaptic currents in medial prefrontal cortex layer II/III pyramidal neurons (Holley *et al*., 2013).

The hippocampus plays a key role in cognitive function, and hippocampal CA1 pyramidal neurons (CA1-PNs) are probably the most widely studied neurons in the mammalian brain. Along with neurons in the subiculum, these cells represent a major output node of this brain area and make synapses with multiple target cells, including a monosynaptic projection to the prefrontal cortex implicated in psychiatric diseases. Additionally, high-resolution structural and functional magnetic resonance imaging studies suggest that the CA1 subregion may be differentially affected in schizophrenia patients (Narr *et al*., 2004; Schobel *et al*., 2009).

Various alterations in synaptic transmission and plasticity in area CA1 of the hippocampus have been reported in different rodent models related to schizophrenia. Deficits in basal synaptic transmission in the SC pathway have been described in the offspring of rats treated with the antimitotic agent methylazoxymethanol during pregnancy (Sanderson *et al*., 2012) and in a maternal immune activation rat model (Oh-Nishi *et al*., 2010). In a transgenic mouse model of the 22q11 deletion syndrome, whilst there were no deficits in basal synaptic transmission, increased excitatory postsynaptic current (EPSC) summation, paired-pulse ratios, and short- and long-term potentiation in the SC pathway have been reported (Earls *et al*., 2010). By contrast, impaired SC LTP has been shown both in rats subchronically dosed with the *N*-methyl-d-aspartate (NMDA) receptor antagonist phencyclidine (Pollard *et al*., 2012), and in a maternal immune activation rat model (Oh-Nishi *et al*., 2010). There is much less information concerning synaptic alterations in DISC1 mouse models. One report describes decreased post-tetanic potentiation with no changes in basal synaptic transmission, paired-pulse ratios or LTP in the SC pathway (Kvajo *et al*., 2008). A more recent study shows decreased short-term potentiation, without changes to basal synaptic transmission or LTP in the mossy fibre pathway (Kvajo *et al*., 2011).

Studies in different mouse models related to schizophrenia have also described alterations to a variety of intrinsic neuronal properties in the hippocampus. Examples include decreased input resistance (*R*_i_) and excitability in granule cells of the dentate gyrus in a DISC1 transgenic mouse model (Kvajo *et al*., 2011), depolarized resting membrane potential (RMP), increased action potential (AP) firing frequency and decreased AP width in CA2/3 stratum oriens interneurons following chronic infusion of picrotoxin into the basolateral amygdala of rats (Gisabella *et al*., 2009), and depolarized RMP and increased excitability of CA1-PNs following systemic administration of the NMDA receptor antagonist MK-801 in mice (Kehrer *et al*., 2007).

To date, no thorough investigations of intrinsic and synaptic properties of CA1-PNs in DISC1 transgenic mouse models have appeared. To address this issue we performed the study detailed here, based on the DISC1_tr_ mouse model developed by Shen *et al*. (2008). These mice express two copies of truncated mouse *Disc1* closely mimicking the situation in the Scottish family with the *Disc1* translocation. These mice are reported to display a variety of schizophrenia-related abnormalities including enlarged lateral ventricles, reduced cerebral cortex volume, and reduced counts of parvalbumin-positive interneurons in the hippocampus and medial prefrontal cortex. Behaviourally, an impairment in conditioning of latent inhibition and ‘depressive-like’ behaviours were described (Shen *et al*., 2008). Our results presented here represent the first description of the neurophysiological consequences of transgenic expression of DISC1_tr_ in this mouse model.

## Materials and methods

As described by Shen *et al*. (2008), DISC1_tr_ transgenic mice were originally generated with a bacterial artificial chromosome containing a truncated form of *Disc1* which encodes the first eight exons. The original line was in a mixed genetic background of CBA/CaCrl and C57BL/6JCrl. Progenies, which were confirmed free of the *Nnt* and *Snca* mutations associated with C57BL/6JCrl and C57BL/6J Harlan substrains, respectively, were backcrossed with mutation-free mice of C57BL/6JRccHsd for nine generations, resulting in DISC1_tr_ hemi mice prior to project initiation. C57BL/6JRccHsd mice were continuously used in all subsequent breeding to generate experimental DISC1_tr_ hemi mice and wild-type (WT) littermate controls.

Male DISC1_tr_ hemi mice and WT littermates were bred and aged at the University of Strathclyde, and subsequently shipped to the University of Bristol by road and housed singly on a 12: 12-h light/dark cycle with *ad libitum* access to food and water. All procedures on experimental animals were approved by local ethical approval at the University of Bristol. Furthermore, all such work was carried out in compliance with UK Home Office regulations as set out in the Animals (Scientific Procedures) Act 1986 and consequently were also in accordance with the European Communities Council Directive of 24 November 1986 (86/609/EEC).

Two cohorts of DISC1_tr_ and WT littermates were employed during this study. The first cohort was used for studies of intrinsic and synaptic properties employing patch-clamp methods and consisted of mice aged ˜3.8 months of age (WT mean age 3.8 ± 0.1 months, range 2.7–4.6 months, *n* = 14 mice; DISC1_tr_ mean age 3.7 ± 0.1 months, range 2.7–4.2 months, *n* = 15 mice). The second cohort was used for studies of synaptic plasticity with extracellular field potential recording and involved mice aged ˜8.7 months (WT mean age 8.7 ± 0.1 months, range 8.2–9.1 months, *n* = 11 mice; DISC1_tr_ mean age 8.7 ± 0.1 months, range 8.2–9.0 months, *n* = 10 mice).

Horizontal hippocampal slices were prepared, and patch-clamp and extracellular field recordings were made at ˜34 °C from area CA1 as described previously (Clement *et al*., 2009; Brown *et al*., 2011; Randall *et al*., 2012) and summarized below.

### Preparation of brain slices

Animals were killed by cervical dislocation and brains were quickly removed and placed into ice-cold sucrose-based artificial slicing solution comprising (in mm): sucrose, 189; d-glucose, 10; NaHCO_3_, 26; KCl, 3; MgSO_4_, 5; CaCl_2_, 0.1; NaH_2_PO_4_, 1.25. Horizontal slices of ventral hippocampus of thickness 300 μm were prepared using a Leica VT1200 vibratome (Leica Microsystems, Milton Keynes, UK). Following their preparation slices were transferred to normal artificial cerebrospinal fluid (aCSF) and incubated at 35–37 °C for 30 min, following which they were kept at room temperature until use. The composition of the standard aCSF was (mm): NaCl, 124; KCl, 3; NaHCO_3_, 26; CaCl_2_, 2; NaH_2_PO_4_, 1.25; MgSO_4_, 1; d-glucose, 10; the solution was equilibrated with 95% O_2_ and 5% CO_2_.

### Single cell patch-clamp recording

For recording, slices were transferred to a submersion-type chamber mounted on an Olympus BX51 WI upright microscope (Scientifica, Uckfield, UK) equipped with infrared differential interference contrast optics to enable visual identification of neurons. Here slices were continuously perfused (˜2.5 mL/min) with normal aCSF and maintained at ˜33 °C. Pharmacological agents were applied via the perfusion system.

Recordings were made using 3–5 MΩ fire-polished glass microelectrodes filled with one of two internal solutions: a K-gluconate-based solution for current-clamp experiments and a CsCl-based solution for voltage-clamp recordings. The pairing of aCSF and pipette solutions produced liquid junction potential errors which were corrected for arithmetically. Signals were amplified using a MultiClamp 700B amplifier (Molecular Devices, Union City, CA, USA), digitized using an Axon Digidata 1440a data acquisition board (Molecular Devices) and stored on a personal computer using pClamp10.2 software (Molecular Devices).

Whole-cell current-clamp recordings were made using a K-gluconate-based internal solution containing (in mm): K-gluconate, 140; NaCl, 10; HEPES, 10; EGTA, 0.2; Na-GTP, 0.3; Mg-ATP, 4; pH 7.3. Current-clamp recordings were made using the bridge circuit of the amplifier to allow faithful voltage following. Recordings of intrinsic and AP properties were lowpass filtered at 10 kHz and digitized at 100 kHz. Sinusoidal current (ZAP current) recordings of intrinsic resonance were lowpass filtered at 0.5 kHz and digitized at 1 kHz. Recordings of synaptic responses in current-clamp were lowpass filtered at 4 kHz and digitized at 20 kHz.

To measure subthreshold, AP and excitability properties, a series of 500-ms square-wave current injection steps (−100 to +300 pA in 50-pA increments) were applied to cells. A sinusoidal current (ZAP current) with constant amplitude and linearly increasing frequency (1–20 Hz over 30 s) was used to characterize resonance properties. This was performed at three different pre-stimulus membrane potentials as these properties are inherently voltage-dependent (Hu *et al*., 2002). To examine fast after-spike potentials, single AP(s) were elicited with a strong but brief (2 nA, 2 ms) current injection.

To measure properties of synaptic responses in CA1-PNs, postsynaptic potentials were evoked by delivering brief (0.1 ms) electrical stimulation to tungsten bipolar stimulating electrodes (FHC Inc., Bowdoin, ME, USA) connected to constant-current isolated stimulator boxes (Digitimer, Welwyn Garden City, UK). Stimulating electrodes were placed in stratum radiatum to stimulate the SC pathway, and in stratum lacunosum moleculare to stimulate the temporoammonic (TA) pathway. Measurements of excitatory and inhibitory postsynaptic potential (EPSP and IPSP) summation were made by delivering trains of six stimuli at different frequencies (5–100 Hz). Stimulus intensity was adjusted to elicit a first EPSP of approximately 4–5 mV (SC) or 0.5–1 mV (TA) in amplitude and was kept constant throughout the stimulus trains.

Whole-cell voltage-clamp recordings were used to specifically measure AMPA (α-amino-3-hydroxy-5-methyl-4-isoxazolepropionic acid) and NMDA currents in the SC pathway using a CsCl-based internal solution containing (in mm): CsCl, 130; NaCl, 5; HEPES, 10; EGTA, 0.2; Na-GTP, 0.3; Mg-ATP, 4; QX314-Cl, 5; pH 7.3. As for current-clamp recordings of synaptic potentials, SC EPSCs were evoked with a stimulating electrode placed in *stratum radiatum*. To block inhibitory postsynaptic currents (IPSCs), which could contaminate evoked excitatory responses, the γ-aminobutyric acid (GABA)_A_ receptor antagonist gabazine (5 μm) was included in the normal aCSF. Area CA3 was removed from the slice to prevent epileptiform discharges that can occur in hippocampal slices in the absence of GABA receptor-mediated inhibition.

Input–output curves were constructed at a holding potential of −80 mV by incrementally increasing stimulus intensity and recording the evoked response (5–40 μA). For subsequent experiments, stimulus intensity was adjusted to elicit an EPSC of approximately 250 pA (at a holding potential of −80 mV) and remained constant throughout. Paired-pulse profiles were constructed at a holding potential of −80 mV by delivering paired stimuli over a range of inter-stimulus intervals. To examine NMDA/AMPA ratios, single stimuli were delivered at holding potentials of −80, +40 and 0 mV. Holding potentials were adjusted to correct for the liquid junction potential error. Voltage-clamp data were lowpass filtered at 4 kHz and digitized at 20 kHz. Series resistance (range 10–20 MΩ) was monitored but not compensated for, and only cells with stable series resistance (<20% change throughout experiment) were included for analysis.

### Extracellular recordings

Area CA3 was removed from the slice before transfer to a submerged chamber (Scientifica) continuously perfused (˜2.5 mL/min) with normal aCSF and maintained at ˜33 °C. Pharmacological agents were added to the aCSF at the concentrations stated and applied to the slice via the perfusion system.

Recordings were made using 2–3 MΩ glass microelectrodes filled with normal aCSF which were fabricated from borosilicate capillary glass (Harvard Apparatus, Edenbridge, UK) using a P-97 Flaming Brown micropipette puller (Sutter Instrument Co., Novato, CA, USA). Stimulating electrodes and recording electrodes were placed in stratum radiatum and stratum lacunosum moleculare. Field excitatory postsynaptic potentials (fEPSPs) were evoked by delivering brief (0.1 ms) electrical stimulation to tungsten bipolar stimulating electrodes (FHC Inc.) connected to a constant-voltage isolated stimulator box (Digitimer). Signals were amplified using a MultiClamp 700 amplifier (Molecular Devices), digitized using an Axon Digidata 1322a data acquisition board (Molecular Devices) and stored on a personal computer using pClamp10.2 software (Molecular Devices). Recordings were lowpass filtered at 10 kHz and digitized at 50 kHz.

The SC and TA pathways were stimulated alternately every 15 s for input–output curves and LTP experiments. Input–output curves were constructed by incrementally increasing stimulus intensity and recording the evoked response (0–12 V in 1-V steps for the SC pathway, and 0–60 V in 5-V steps for the TA pathway). Paired-pulse profiles were constructed by delivering paired stimuli over a range of inter-stimulus intervals at a stimulus intensity that elicited an approximately half-maximal response.

Long-term potentiation was induced using a theta burst stimulation (TBS) protocol. A baseline period of at least 20 min stable fEPSPs was recorded at a stimulation intensity that elicited an approximately half-maximal response. Stimulation intensity remained constant throughout the experiment, including during the TBS protocol. Following the baseline period, the TBS protocol was delivered to one pathway and consisted of five bursts (10 stimuli at 100 Hz) at 5 Hz (theta frequency), repeated four times at an interval of 20 s. fEPSPs were then followed for 1 h before TBS stimulation was delivered to the other pathway and fEPSPs were followed for another hour. The order in which the pathways were stimulated with the TBS protocol was alternated between experiments.

Data analyses were carried out using pClamp10.2, Excel and custom-written routines in MatLab. A detailed description of the algorithms used for analysis of CA1-PNs can be found in Kerrigan *et al*. (2013). spss was used to carry out statistical analyses.

## Results

### Intrinsic properties of CA1-PNs

**Figure 1:**
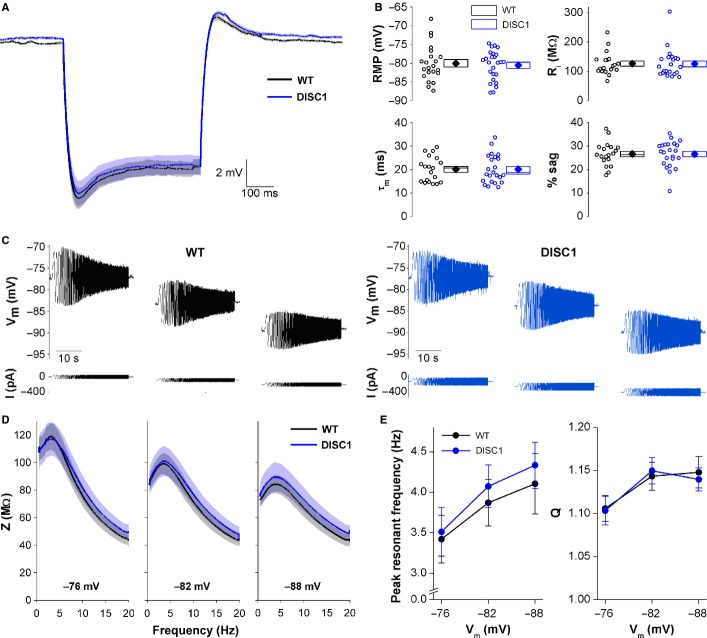
Passive membrane and resonance properties of CA1-PNs are not altered in DISC1_tr_ mice. (A) Mean voltage traces of responses to 500 ms, -100 pA square wave current injection applied at a fixed pre-stimulus potential of −80 mV. The shaded areas represent the SEM. *n* = 19 WT and 23 DISC1_tr_ CA1-PNs from nine WT and nine DISC1_tr_ mice. (B) Scatter plot showing RMP, *R*_i,_ τ_M_ and percentage sag from all recorded neurons (open symbols). Mean (filled symbols), SEM (box) and median (central line) are shown to the right. (C) Representative traces from WT (black) and DISC1_tr_ (blue) CA1-PNs showing the ZAP current protocol (bottom) and corresponding voltage responses (top) at −76, −82 and −88 mV. (D) Mean impedance (*Z*) profiles at the three membrane potentials tested. The shaded areas represent the SEM. (E) Mean ± SEM peak resonant frequency and *Q* values (strength of resonance) against membrane potential. *n* = 17 WT and 16 DISC1_tr_ CA1-PNs from nine WT and nine DISC1_tr_ mice.

*Passive membrane properties, and sag and rebound potentials are genotype-independent*. Passive subthreshold membrane properties of CA1-PNs were recorded from 13 slices from nine WT mice and 10 slices from nine DISC1_tr_ mice of 3–4 months of age. There was no difference in the RMP recorded in the first minute after gaining whole cell access (Fig.1B, Table1; WT −80.0 ± 1.0 mV; DISC1 −80.5 ± 0.8 mV; *P* = 0.7; unpaired, two-tailed Student’s *t* test; *n* = 19 WT and 23 DISC1_tr_ CA1-PNs). Because other passive membrane properties including *R*_i_, membrane time constant (τ_M_) and sag upon hyperpolarization exhibit some voltage-dependence, after determination of RMP, CA1-PNs were held at a set pre-stimulus membrane potential of −80 mV by employing an appropriate level of steady-state current injection. A 500-ms, 100-pA hyperpolarizing square-wave current injection step was then applied. The mean voltage responses to this stimulus obtained from the two genotypes are shown in Fig.1A. From such voltage responses we extracted five additional measurements on a cell by cell basis (see Kerrigan *et al*., 2013). Of these, the Shapiro–Wilk test for normality indicated that RMP, τ_M_ and percentage sag measurements were normally distributed, whereas *R*_i_, negative peak and rebound were not normally distributed (*R*_i_ WT *P* < 0.05, DISC1 *P* < 0.001; negative peak WT *P* < 0.05, DISC1 *P* < 0.001; rebound WT *P* < 0.05, DISC1 *P* < 0.001). Comparison of these measures between genotypes using appropriate statistical tests (unpaired, two-tailed Student’s *t* test for normally distributed data, and independent samples Mann–Whitney *U* test for non-normally distributed data) revealed no genotype-related differences in any of these subthreshold passive membrane properties (Fig.1B, Table1).

**Table 1:** Passive membrane properties (mean ± SEM) of CA1-PNs from 3- to 4-month-old DISC1_tr_ and WT littermates

	WT (*n*=19)	DISC1_tr_ (*n*=23)	*P*
RMP (mV)	−80.0 ± 1.0	−80.5 ± 0.8	0.7
*R*_i_ (MΩ)	127 ± 9	126 ± 10	0.6
τ_M_ (ms)	20.2 ± 1.2	20.2 ± 1.3	1
Sag (%)	26.7 ± 1.2	26.6 ± 1.2	1
Max –ve deflection (mV)	11.8 ± 0.8	11.7 ± 0.9	0.5
Rebound on repol (mV)	2.3 ± 0.2	2.3 ± 0.1	1

Resting membrane potential (RMP) was measured straight after entering whole cell recording mode, whereas other parameters were measured using 500-ms, −100-pA current step applied from a set pre-stimulus membrane potential of −80 mV. *R*_i_, input resistance; τ_M_, membrane time constant.

The ZAP (impedance amplitude profile) method was used to characterize the subthreshold resonance properties of CA1-PNs in DISC1_tr_ mice at three different pre-stimulus membrane potentials (−76, −82 and −88 mV). Representative traces from WT and DISC1_tr_ CA1-PNs are shown in Fig.1C and the mean impedance (*Z*) profiles at each potential are shown in Fig.1D. There was a significant effect of voltage on both the peak resonant frequency and strength of resonance (*Q*) (Fig.1E; peak frequency *F* = 27.9, *P* < 0.001; *Q F* = 35.4, *P* < 0.001; repeated-measures anova). As the resonance properties of neurons are related to their passive membrane properties and sag (Hu *et al*., 2002; Biel *et al*., 2009), and these were not altered in DISC1_tr_ CA1-PNs, it was not surprising that there was no significant effect of genotype on peak resonant frequency or *Q* values (Fig.1E; peak frequency *F* = 0.2, *P* = 0.7; *Q F* = 0.01, *P* = 0.9; repeated-measures anova; *n* = 17 WT and 16 DISC1_tr_ CA1-PNs).

### Action potential properties are genotype-independent

Similar to our measurements of passive membrane properties (Fig.1), AP properties were measured from a set pre-stimulus membrane potential of −80 mV. Our analysis focused on the first AP evoked by a 300-pA depolarizing current injection. This level of depolarizing current stimulus produced at least one AP in all CA1-PNs examined (see below). Statistical tests for normality showed that all AP properties were normally distributed. Analysis of AP peak, threshold, width and maximum rate of rise (unpaired, two-tailed Student’s *t* test) showed that these properties were not dependent upon genotype, as shown in Table2.

**Table 2:** Details of AP properties (mean ± SEM) of CA1-PNs from 3- to 4-month-old DISC1_tr_ and WT littermates

	WT (*n*=19)	DISC1_tr_ (*n*=23)	*P*
AP peak (mV)	31.1 ± 1.3	31.7 ± 1.3	0.8
AP width at −15 mV (ms)	0.86 ± 0.02	0.88 ± 0.02	0.5
AP threshold (mV)	−57.1 ± 0.8	−56.8 ± 1.0	0.8
AP max. RoR (mV/ms)	443 ± 17	452 ± 19	0.7

AP width was measured at −15 mV. Threshold was defined as the voltage when the rate of rise (RoR) of the AP upstrokes first exceeded 15 mV/ms.

### DISC1_tr_ CA1-PNs have a greater initial burst of APs upon weak depolarization

**Figure 2:**
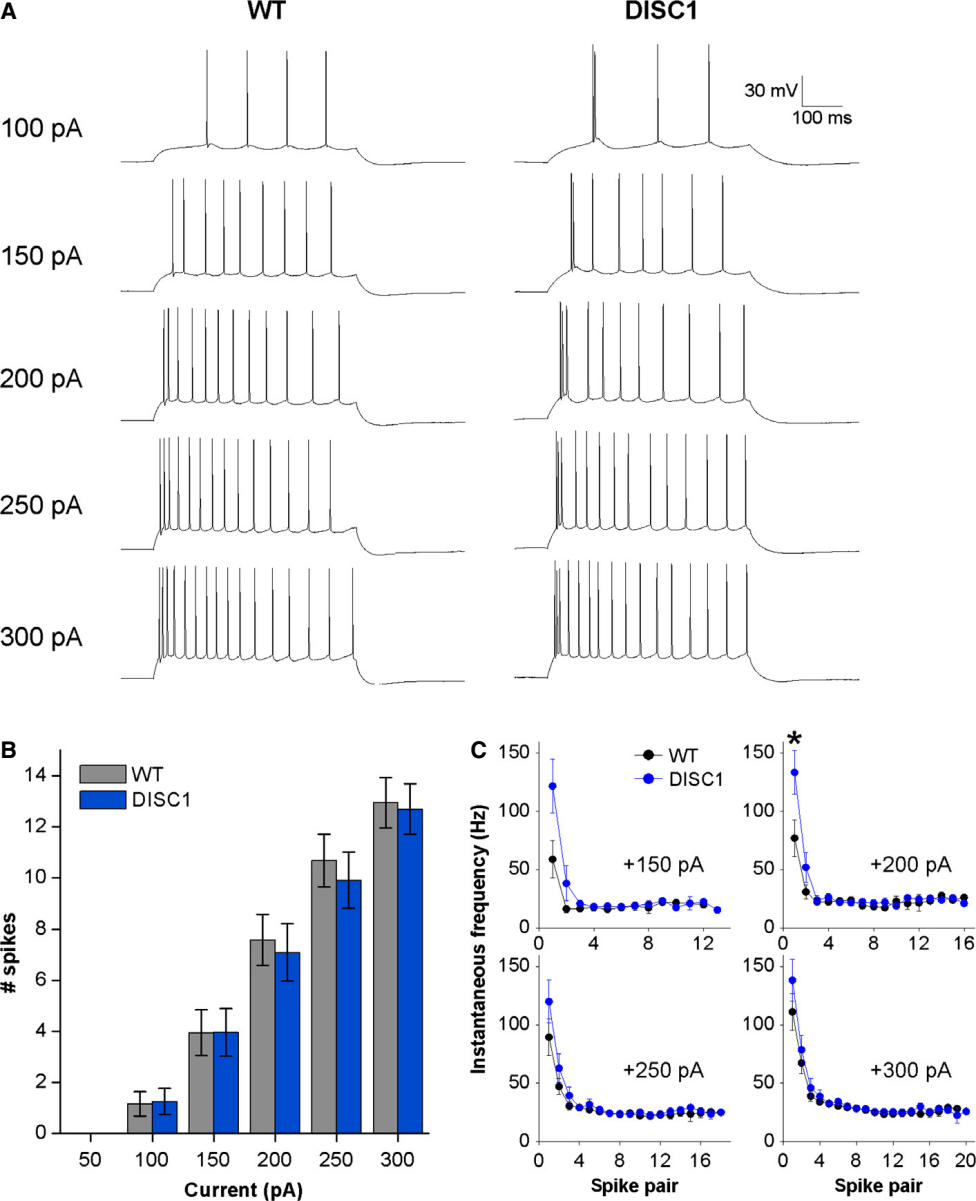
DISC1_tr_ CA1-PNs display a more prominent initial burst of APs upon mild depolarization. (A) Representative traces showing AP firing patterns in response to square-wave depolarizing current injections of increasing magnitude. (B) The number of APs fired at each current injection step was not different between WT and DISC1_tr_ CA1-PNs. (C) Instantaneous frequency plots reveal a more prominent initial burst of APs in response to mild, but not strong, depolarization in DISC1_tr_ CA1-PNs compared with WT. In B and C, data are mean ± SEM. *P < 0.05; independent-samples Mann–Whitney *U* test; *n* = 19 WT and 23 DISC1_tr_ CA1-PNs from nine WT and nine DISC1_tr_ mice.

To examine patterns of AP firing in CA1-PNs, 500-ms depolarizing current injections of various magnitudes were applied to the cells from a set potential of −80 mV. Representative responses to these current injections from WT and DISC1_tr_ CA1-PNs are shown in Fig.2A. The number of APs fired at each stimulus intensity was not different between DISC1_tr_ and WT CA1-PNs (Fig.2B; *F* = 0.05, *P* = 0.8; repeated-measures anova; *n* = 19 WT and 23 DISC1_tr_ CA1-PNs).

Under these recording conditions, and when activated by a maintained suprathreshold current step, murine CA1-PNs typically display an initial high-frequency burst of APs followed by accommodation to a lower firing frequency of ˜25–40 Hz (Brown *et al*., 2011; Randall *et al*., 2012; Kerrigan *et al*., 2013). Furthermore, the frequency of APs within the initial burst tends to increase with increased stimulus intensity. This typical firing pattern was observed in WT CA1-PNs, as can be seen in the instantaneous frequency plots in Fig.2C. In DISC1_tr_ CA1-PNs, however, the initial high-frequency burst was similar at all stimulus intensities examined (Fig.2C), and consequently following mild depolarizing stimuli (150, 200 pA) DISC1_tr_ CA1-PNs had a more prominent initial burst of APs than WT CA1-PNs. Comparison of the frequency of the first spike pairs revealed a trend towards higher frequency at a stimulus intensity of 150 pA (*P* = 0.08) and a significantly higher frequency at 200 pA (*P* = 0.04; independent samples Mann–Whitney *U* test). This difference was not evident at stronger depolarizing stimuli (Fig.2C; 250 pA, *P* = 0.3; 300 pA, *P* = 0.3). This increased burstiness is similar to observations we have made in the amyloid beta-overproducing mouse lines PSAPP (Brown *et al*., 2011) and PDAPP (Kerrigan *et al*., 2013).

### Spike after-potentials are genotype-independent

**Figure 3:**
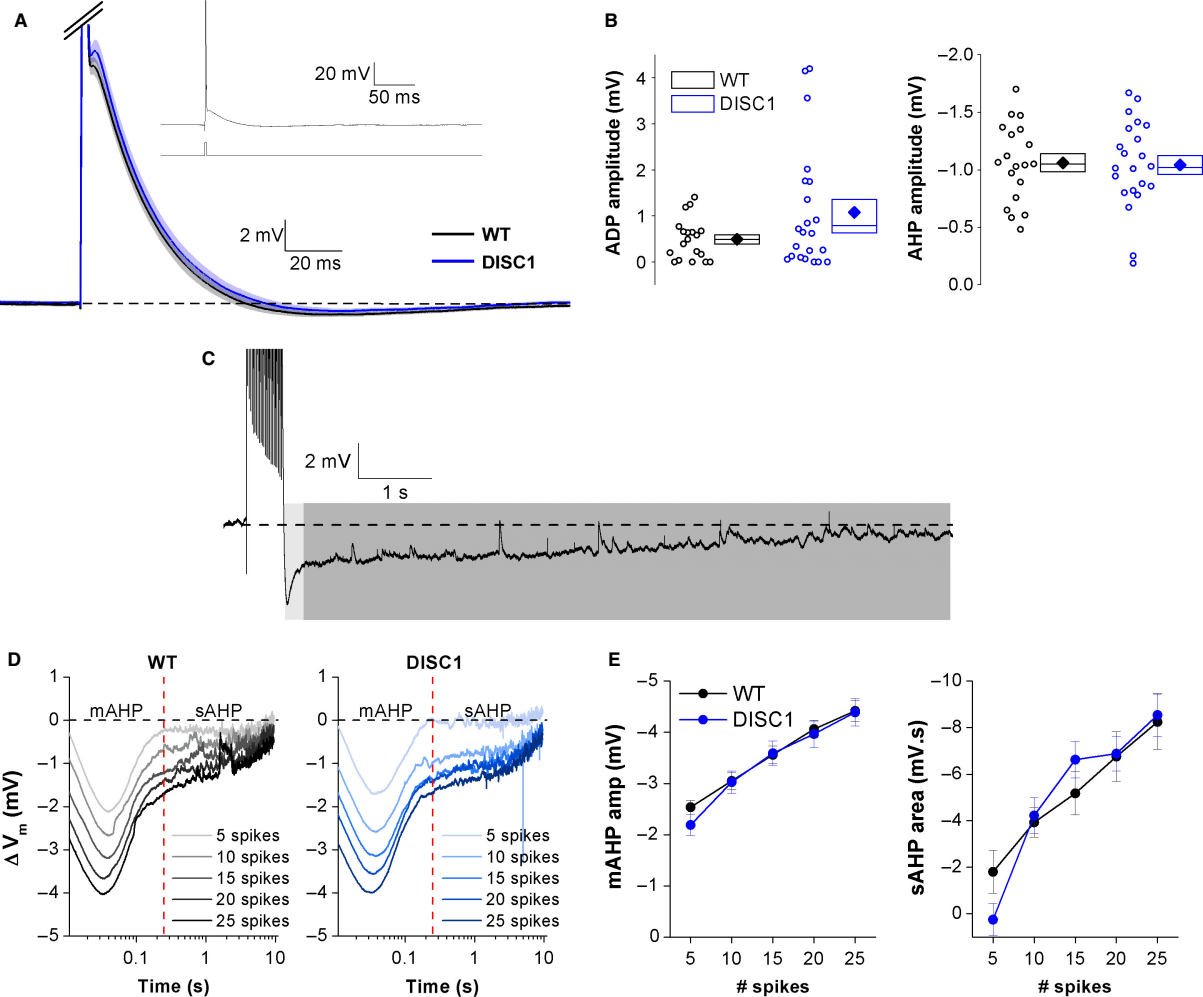
After-spike potentials are genotype-independent. (A) Mean voltage traces following a single AP (truncated for clarity) elicited from a fixed potential of −80 mV. The shaded areas represent the SEM. The inset graph shows the current protocol used (bottom) and a full-scale representative voltage response (top). (B) Scatter plot showing ADP and AHP amplitudes from all recorded neurons (open symbols). Mean (filled symbols), SEM (box) and median (central line) are shown on the right. *n* = 19 WT and 22 DISC1_tr_ CA1-PNs from nine WT and nine DISC1_tr_ mice. (C) Representative trace showing the mAHP (light grey) and sAHP (dark grey) elicited following a train of 20 APs fired at 50 Hz. The APs are truncated for clarity. (D) Mean voltage traces following 5–25 APs (lightest to darkest) elicited at a frequency of 50 Hz. (E) mAHP amplitude and sAHP area both increase with increasing number of APs, although neither is significantly different between WT and DISC1_tr_ CA1-PNs. Data are mean ± SEM. *n* = 18 WT and 21 DISC1_tr_ CA1-PNs from nine WT and nine DISC1_tr_ mice.

Spike after-potentials are important in the control of AP firing patterns. Fast after-spike depolarization potentials (ADPs) play an important role in the initial high-frequency AP burst observed upon depolarization of hippocampal pyramidal cells (Jensen *et al*., 1996; Yue & Yaari, 2004; Brown & Randall, 2009). Because DISC1_tr_ CA1-PNs had an enhanced initial burst frequency, the ADP was examined following a single AP elicited with a strong but brief (2 nA, 2 ms) current injection (Fig.3A, inset graph). Analysis revealed a trend towards a larger ADP in DISC1_tr_ CA1-PNs, which was not significant (Fig.3B; *P* = 0.2; independent-samples Mann–Whitney *U* test; *n* = 19 WT and 22 DISC1_tr_ CA1-PNs), but could contribute to the increased initial burst frequency described above.

After-spike hyperpolarizing potentials (AHPs) can be divided into fast, medium and slow components, each of which are reported to be mediated by a variety of voltage-gated and/or Ca^2+^-dependent K^+^ channels. AHPs are important for various neurophysiological properties, most notably spike frequency accommodation (Madison & Nicoll, 1984; Storm, 1989). In our recordings, a small medium AHP (mAHP) can be observed following a single spike, as shown in the mean traces plotted in Fig.3A. The amplitude of the small mAHP observed following a single AP was not different between WT and DISC1_tr_ CA1-PNs (Fig.3B).

When CA1-PNs are induced to fire a train of spikes as shown in Fig.3C, a larger mAHP (light grey shaded area) is seen and a slow AHP (sAHP; darker grey shaded area) also becomes apparent. The amplitude of the mAHP and area of the sAHP was measured following trains of 5–25 APs elicited at a frequency of 50 Hz. Figure3D shows mean traces from WT and DISC1_tr_ CA1-PNs plotted on a log time scale for clarity. This shows that both components become significantly larger with increasing number of spikes (mAHP amplitude *F* = 107.7, *P* < 0.001; sAHP area *F* = 48.2, *P* < 0.001; repeated-measures anova). Cross-genotype comparison revealed that mAHP amplitude and sAHP area were almost identical in the two groups (Fig.3E; mAHP amplitude *F* = 0.07, *P* = 0.8; sAHP area *F* = 0.03, *P* = 0.9; repeated-measures anova; *n* = 18 WT and 21 DISC1_tr_ CA1-PNs).

### Patch-clamp analysis of synaptic inputs to CA1-PNs

CA1-PNs receive multiple glutamatergic inputs, of which the SC and TA pathways are particularly prominent. The former pathway has been very widely studied, and indeed is probably the most widely studied pathway in the mammalian CNS, whereas the TA pathway has received much less attention, especially in disease models. Both pathways can be easily studied in the same hippocampal slice, an approach we employed to examine the SC and TA pathways in DISC1_tr_ mice. First, whole-cell recordings were made to study EPSP and IPSP summation in both pathways in individual CA1-PNs. Secondly, extracellular field recordings were used to investigate basal synaptic transmission, paired-pulse facilitation and LTP in populations of CA1 neurons in both pathways as this method circumvents any issues arising from dialysis of intracellular components that confound, for example, LTP studies in the standard whole-cell patch-clamp configuration.

### EPSP and IPSP summation in the SC pathway

**Figure 4:**
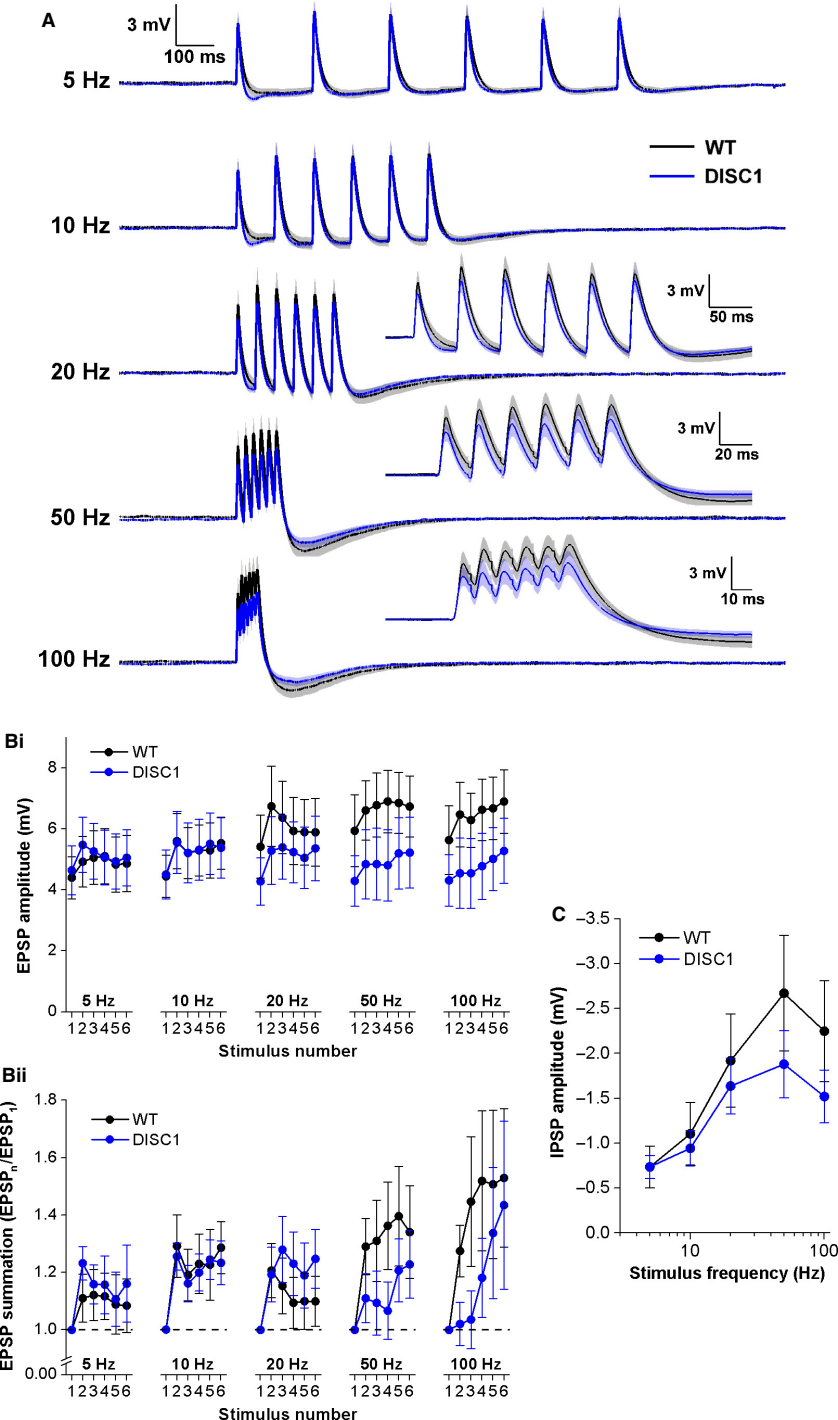
Schaffer collateral EPSP and IPSP summation. (A) Mean voltage responses to trains of six stimuli delivered at 5, 10, 20, 50 and 100 Hz from WT (black) and DISC1_tr_ (blue) CA1-PNs. Inset graphs show responses on a larger time scale for clarity. The shaded areas represent the SEM. (B) Absolute amplitudes (i) and amplitudes normalized to the first response (ii) for each EPSP in the train. (C) Amplitude of the IPSP at the end of each train plotted against stimulus frequency. In B and C, data are mean ± SEM. *n* = 14 WT and 14 DISC1_tr_ CA1-PNs from nine WT and nine DISC1_tr_ mice.

The relationship between synaptic response and stimulus frequency was examined for short stimulus trains. The group mean voltage responses from both genotypes, at each stimulus frequency tested, are shown in Fig.4A. To examine EPSPs, the peak level of positivity following each stimulus was measured relative to pre-stimulus baseline membrane potential, which was set to −80 mV using appropriate steady-state current injection. Figure4B shows that at all frequencies tested, EPSPs summated throughout the trains [presented as both absolute amplitudes (Fig.4Bi) and normalized to the amplitude of the first EPSP in each train (Fig.4Bii)]. Summation was greater at higher frequencies but also more variable. A trend toward decreased EPSP summation in DISC1_tr_ CA1-PNs was observed at 50 and 100 Hz, but this was not significantly different from WT CA1-PNs (Fig.4B; 100-Hz normalized amplitudes *F* = 0.9, *P* = 0.3; repeated-measures anova; *n* = 14 WT and 14 DISC1_tr_ CA1-PNs).

Following the last EPSP in each train, an IPSP was apparent which became larger with increased stimulus frequency (Fig.4C; *F* = 31.8, *P* < 0.001; repeated-measures anova). Comparison between genotypes revealed no differences in IPSP amplitudes (Fig.4C; *F* = 0.9, *P* = 0.4; repeated-measures anova; *n* = 14 WT and 14 DISC1_tr_ CA1-PNs).

**Figure 5:**
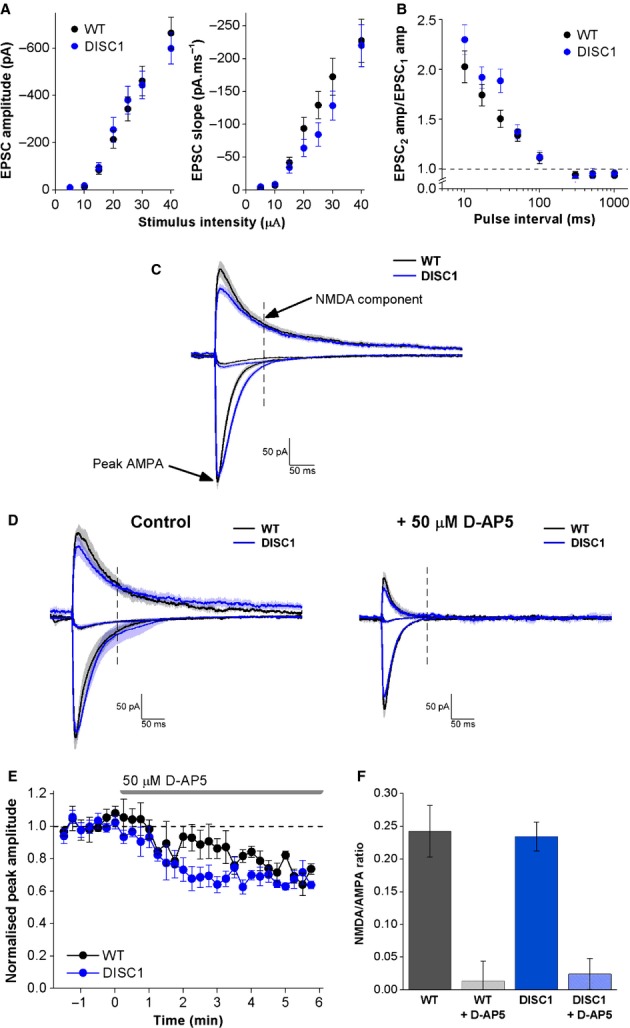
Schaffer collateral NMDA/AMPA ratios are genotype-independent. (A) Peak amplitude and slope measurements for input–output curves recorded at −80 mV. (B) Paired-pulse ratios recorded at −80 mV. (C) Mean traces from all cells showing where measurements of AMPA and NMDA receptor activation were made. (D) Mean traces from a subset of cells treated with 50 μm D-AP5 (*n* = 4 WT and *n* = 5 DISC1 CA1-PNs). (E) Effect of D-AP5 on EPSC peak recorded at −80 mV shows that NMDA receptors make a small contribution to the peak at this membrane potential (*n* = 4 WT and *n* = 5 DISC1 CA1-PNs). (F) NMDA/AMPA ratios from all cells, and cells treated with 50 μm D-AP5 which abolishes the NMDA component. Data in A, B, E and F are mean ± SEM. In C and D, the shaded areas represent the SEM. *n* = 16 WT and 17 DISC1_tr_ CA1-PNs from five WT and six DISC1_tr_ mice.

Changes in AMPA and/or NMDA receptor activation could contribute to differences in EPSP summation. As there was a trend towards decreased SC EPSP summation in DISC1_tr_ CA1-PNs, voltage-clamp recordings were used to investigate AMPA and NMDA receptor-mediated currents in the SC pathway. As described in the Methods, these recordings were made with Cs^+^-based pipette solutions and in the presence of gabazine, a selective GABA_A_ receptor antagonist. Input–output curves were recorded at −80 mV and consequently mainly represent activation of AMPA receptors. The input–output relationships of peak amplitude and slope of EPSCs were unaffected by genotype (Fig.5A; amplitude *F* = 0.002, *P* = 1; slope *F* = 1.0, *P* = 0.3; repeated-measures anova; *n* = 23 WT and 23 DISC1_tr_ CA1-PNs) suggesting that AMPA receptor activation is not altered in DISC1_tr_ CA1-PNs compared with WT. Paired-pulse profiles for EPSCs were also constructed at −80 mV, and facilitation was seen at pulse intervals shorter than 100 ms as shown in Fig.5B. Again, there was no effect of genotype on the paired-pulse profiles (*F* = 2.8, *P* = 0.1; repeated-measures anova; *n* = 20 WT and 19 DISC1_tr_ CA1-PNs) suggesting that presynaptic glutamate release probability is also unaffected by genotype.

To investigate NMDA/AMPA ratios, stimulus intensity was set to evoke an EPSC (at −80 mV) of approximately 250 pA in amplitude and was kept constant throughout the rest of the experiment. Currents were evoked when holding each cell at potentials of −80, +40 and 0 mV. At −80 mV the peak amplitude was measured and represents activation mainly of AMPA receptors. At +40 mV, both AMPA and NMDA receptor activation contribute to the current at peak amplitude, so to gain a measure of NMDA receptor activation only, the mean amplitude at 100–105 ms post-stimulus was calculated. Figure5C shows mean traces from all cells and indicates the measurements used. To confirm that the NMDA region measured represents NMDA receptor activation only and that AMPA receptor activation does not contribute at this time-point, a subset of cells were treated with the NMDA receptor antagonist D-AP5 (50 μm). Figure5D shows the mean responses in this subset of cells before and after D-AP5 application, and confirms that AMPA receptor activation does not contribute to the current evoked at this time-point (the dashed line represents the NMDA time-point). Application of D-AP5 also reduced the peak amplitude of the EPSC recorded at −80 mV by approximately 30% (as shown in the time-course plot of D-AP5 application in Fig.5E), showing that NMDA receptor activation does contribute to the EPSC peak at this potential. Both genotypes were equally affected by D-AP5 treatment (Fig.5E; WT 28 ± 3% reduction; DISC1_tr_ 32 ± 3% reduction; *P* = 0.3; unpaired, two-tailed Student’s *t* test; *n* = 4 WT & 5 DISC1_tr_ CA1-PNs). NMDA/AMPA ratios were calculated for each cell by dividing the NMDA component by the peak AMPA amplitude, and were unaffected by genotype (Fig.5F; *P* = 0.9; unpaired, two-tailed Student’s *t* test; *n* = 16 WT and 17 DISC1_tr_ CA1-PNs). The fact that NMDA/AMPA ratios and the reduction in EPSC peak amplitude at −80 mV following application of D-AP5 were not different between genotypes suggests that NMDA receptor activation is not altered in DISC1_tr_ CA1-PNs compared with WT.

Examination of the mean traces in Fig.5C revealed a potentially decreased EPSC peak at +40 mV and altered decay kinetics at −80 mV in DISC1_tr_ CA1-PNs, although access resistance was significantly higher in DISC1_tr_ CA1-PNs (WT 14.0 ± 0.5 MΩ; DISC1_tr_ 16.5 ± 0.7 MΩ; *P* < 0.01; unpaired, two-tailed Student’s *t* test), which could affect both peak measurements and kinetics, so these observations were not investigated further. The AMPA/NMDA ratio measurements are still valid despite the difference in access resistance between genotypes as this measure is ratiometric and calculated for each individual cell (access resistance was unchanged within each experiment).

### EPSP and IPSP summation in the temporoammonic pathway

**Figure 6:**
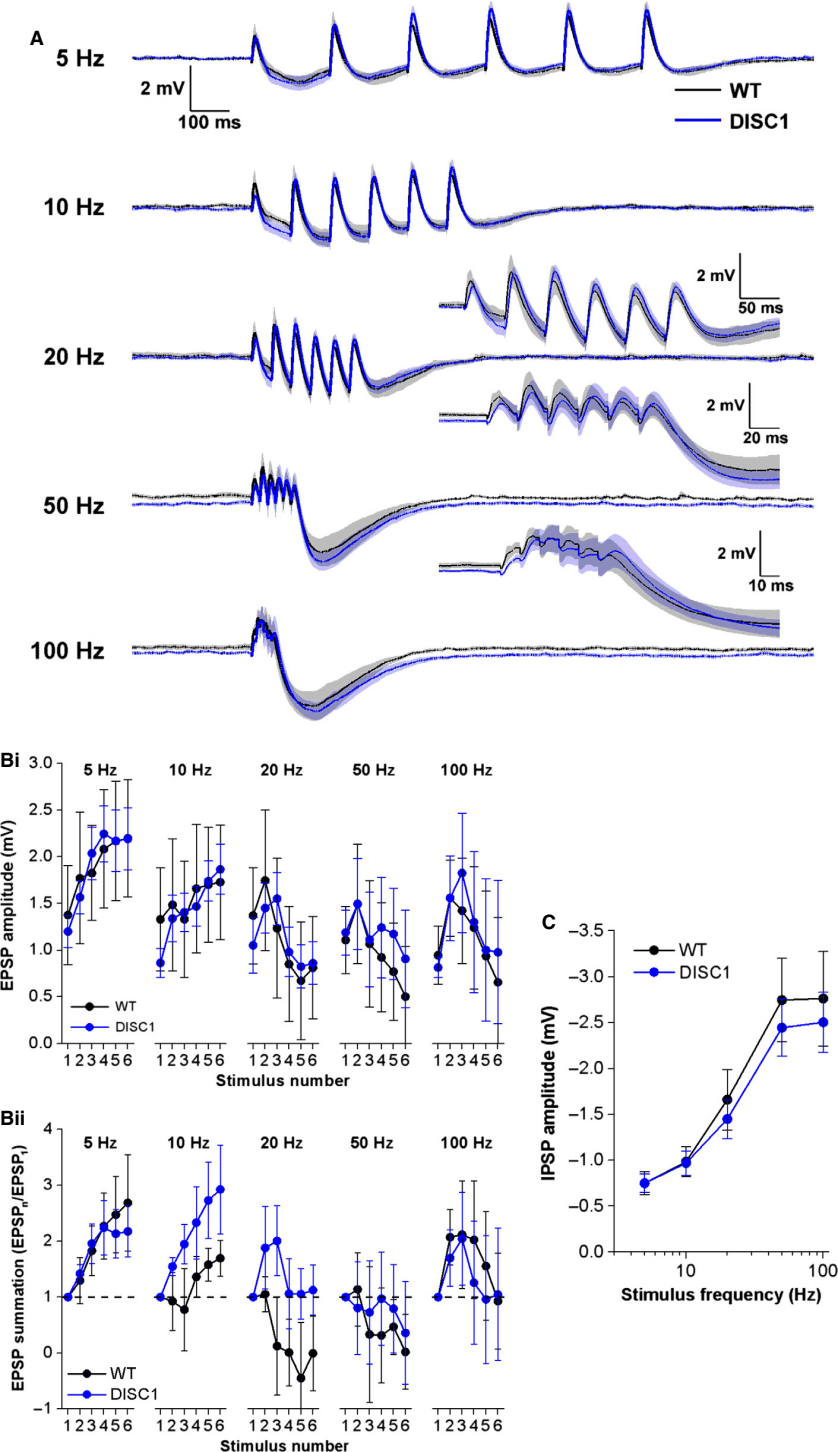
Temporoammonic EPSP and IPSP summation. (A) Mean voltage responses to trains of six stimuli delivered at 5, 10, 20, 50 and 100 Hz from WT (black) and DISC1_tr_ (blue) CA1-PNs. Inset graphs show responses on a larger time scale for clarity. The shaded areas represent the SEM. (B) Absolute amplitudes (i) and amplitudes normalized to the first response (ii) for each EPSP in the train. (C) Amplitude of the IPSP at the end of each train plotted against stimulus frequency. Data are mean ± SEM. *n* = 7 WT and *n* = 8 DISC1_tr_ CA1-PNs from six WT and seven DISC1_tr_ mice.

EPSP and IPSP summation was studied in the TA pathway in the same way as described for the SC pathway, but to stimulate TA postsynaptic potentials, the stimulating electrode was placed in stratum lacunosum moleculare. Only recordings with a first TA EPSP >0.3 mV were included in analysis. The mean responses across cells at each frequency are shown in Fig.6A. EPSP summation was clearly evident at stimulus frequencies of 5 and 10 Hz, but at higher frequencies there was little or no summation throughout the train of stimuli, probably due to the effect of the large, slow IPSP that became apparent at higher frequencies [Fig.6B: absolute amplitudes (i) and normalized to the amplitude of the first EPSP in each train (ii)]. Cross-genotype comparison revealed no differences in EPSP summation at any of the frequencies tested.

The slow IPSP, which is mediated by GABA_B_ receptors (our unpublished observations), became significantly larger with increased stimulus frequency (Fig.6C; *F* = 64.6, *P* < 0.001; repeated-measures anova), although comparison between genotypes revealed no differences in IPSP amplitudes (Fig.6C; *F* = 0.3, *P* = 0.6; repeated-measures anova; *n* = 7 WT and eight DISC1_tr_ CA1-PNs).

### Extracellular analysis of synaptic function and plasticity

Alterations in basal synaptic transmission, paired-pulse facilitation and LTP in area CA1 have been demonstrated in various models of schizophrenia (as described above), so the following experiments were carried out to examine these phenomena in 11 WT and 10 DISC1_tr_ mice which were about 8–9 months of age using extracellular fEPSP recordings.

### SC commissural pathway

**Figure 7:**
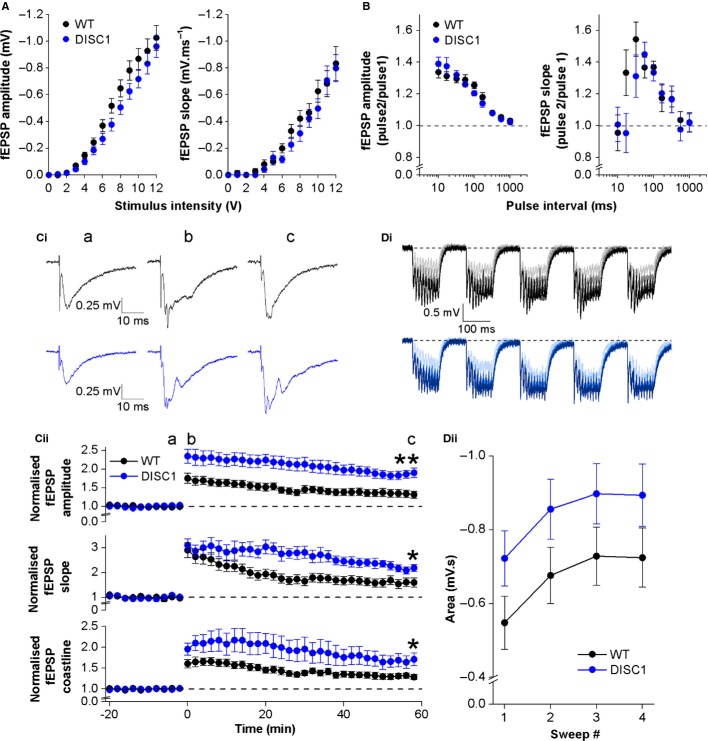
Basal synaptic transmission, paired-pulse profiles and LTP in the Schaffer collateral pathway. (A) Input–output relationships. *n* = 26 WT and 20 DISC1_tr_ slices. (B) Paired-pulse profiles. *n* = 15 WT and 13 DISC1_tr_ slices. (C) TBS-induced LTP. Representative traces from baseline (a), immediately post-TBS (b) and 60 min post-TBS (c) are shown in Ci for WT (black) and DISC1_tr_ (blue) slices. Time-course of fEPSP amplitude, slope and coastline following TBS are shown in Cii. (Di) Representative traces showing responses to the TBS protocol for WT (black) and DISC1_tr_ (blue). The four sweeps are superimposed [first sweep (lightest trace) to last sweep (darkest trace)]. (Dii) Area below baseline for each of the four sweeps during the TBS protocol. Data are mean ± SEM. *P < 0.05, **P < 0.005; unpaired, two-tailed Student’s *t* test; *n* = 13 WT and nine DISC1_tr_ slices.

In agreement with input–output curves in the SC pathway measured with voltage-clamp of individual CA1-PNs (Fig.5A), extracellular recordings of population EPSPs in this pathway revealed no difference in basal synaptic transmission between WT and DISC1_tr_ slices (Fig.7A; fEPSP amplitude *F* = 1.2, *P* = 0.3; fEPSP slope *F* = 0.5, *P* = 0.5; repeated-measures anova; *n* = 26 WT and 20 DISC1_tr_ slices). Analysis of paired-pulse fEPSP amplitudes with respect to baseline showed facilitation at all stimulation intervals shorter than 500 ms (Fig.7B, left). However, at intervals of 10 and 17 ms, the second EPSP was elicited before the previous response had returned to baseline and so the first response contributed to the amplitude of the second response. Slope measurements (Fig.7B, right) are not so severely contaminated and are probably more representative of the true facilitation in this pathway. Paired-pulse fEPSP slope measurements revealed a bell-shaped relationship, with greatest facilitation at intervals between 30 and 100 ms. Cross-genotype comparisons showed no difference in either measurement, confirming the outcome of voltage-clamp analysis (Fig.5B) and suggesting that presynaptic glutamate release is not altered in this pathway in DISC1_tr_ mice (fEPSP_2_/fEPSP_1_ amplitude *F* < 0.001, *P* = 1; fEPSP_2_/fEPSP_1_ slope *F* = 1.5, *P* = 0.2; repeated-measures anova; *n* = 15 WT and 13 DISC1_tr_ slices).

Activity-dependent LTP can be induced in the SC pathway in a number of ways. In this study we chose to use a TBS protocol which has some relevance to the firing patterns of CA3 neurons (the presynaptic cell in the SC pathway) *in vivo* (see Materials and methods). Figure7C plots baseline-normalised fEPSP amplitude and slope for a 20-min baseline period and 60 min post-TBS. In both genotypes, TBS was able to induce significant LTP (WT fEPSP amplitude *t* = 3.4, *P* < 0.005; WT fEPSP slope *t* = 3.5, *P* < 0.005; DISC1_tr_ fEPSP amplitude *t* = 8.4, *P* < 0.001; DISC1_tr_ fEPSP slope *t* = 9.8, *P* < 0.001; one-sample, two-tailed Student’s *t* test), while comparison of the magnitude of LTP between genotypes revealed significantly greater potentiation in DISC1_tr_ slices. In WT slices, fEPSP amplitude was on average 1.3 ± 0.1 times greater and slope was 1.6 ± 0.2 times greater than baseline 1 h post-TBS, whereas in DISC1_tr_ slices fEPSP amplitude was 1.9 ± 0.1 times greater and slope was 2.2 ± 0.1 times greater than baseline at the same time-point. Statistical comparison demonstrated that these differences were significant (fEPSP amplitude *t* = 3.5, *P* < 0.005; fEPSP slope *t* = 2.5, *P* < 0.05; unpaired, two-tailed Student’s *t* test; *n* = 13 WT and 9 DISC1_tr_ slices).

Closer examination of individual traces showed that fEPSPs following the TBS conditioning protocol appeared to be more ‘epileptic’ in DISC1_tr_ slices (Fig.7Ci). To quantify this fEPSP, ‘coastlines’ were also measured. Coastline analysis revealed significantly greater (and more variable) potentiation in DISC1_tr_ slices compared with WT (Fig. 7Cii; *t* = 2.5, *P* < 0.05; unpaired, two-tailed Student’s *t* test; *n* = 13 WT and 9 DISC1_tr_ slices).

Because LTP was enhanced in DISC1_tr_ slices, the area below baseline during the TBS LTP-induction protocol was measured, as this could indicate differences in postsynaptic drive during the conditioning stimulus, which in turn could potentially underlie changes in LTP. Figure7D shows representative traces and pooled areas for each of the four repetitions (20-s inter-sweep interval) of the TBS protocol. Area below baseline significantly increased with successive sweeps (Fig.7Dii; *F* = 26.7, *P* < 0.001; repeated-measures anova), and whilst area below baseline was greater in DISC1_tr_ slices than in WT, this was not quite significant (*F* = 2.4, *P* = 0.1; repeated-measures anova; *n* = 13 WT and 9 DISC1_tr_ slices).

### TA pathway

**Figure 8:**
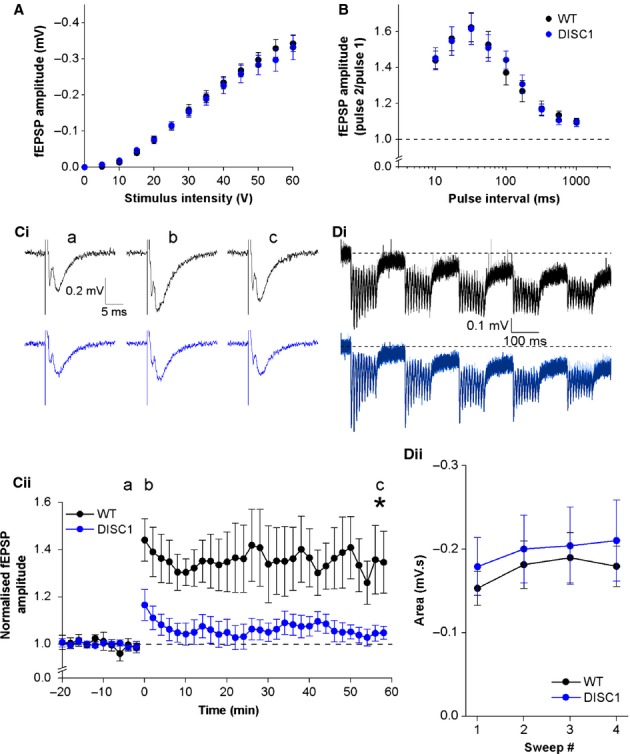
Basal synaptic transmission, paired-pulse profiles and LTP in the temporoammonic pathway. (A) Input–output relationships. *n* = 26 WT and 20 DISC1_tr_ slices. (B) Paired-pulse profiles. *n* = 12 WT and 9 DISC1_tr_ slices. (C) TBS-induced LTP. Representative traces from baseline (a), immediately post-TBS (b) and 60 min post-TBS (c) are shown in Ci for WT (black) and DISC1_tr_ (blue) slices. Time-course of fEPSP amplitude following TBS is shown in Cii. (Di) Representative traces showing responses to the TBS protocol for WT (black) and DISC1_tr_ (blue). The four sweeps are superimposed [first sweep (lightest trace) to last sweep (darkest trace)]. (Dii) Area below baseline for each of the four sweeps during the TBS protocol. Data are mean ± SEM. *P < 0.05; unpaired, two-tailed Student’s *t* test; *n* = 13 WT and 11 DISC1_tr_ slices.

In the TA pathway, fEPSPs were smaller and required stronger stimulation to elicit responses than in the SC pathway. Slope measurements of TA fEPSPs were very variable, probably due to the small size of the responses, and so only fEPSP amplitude data are described below. Similar to the SC pathway, input–output curves in the TA pathway revealed no difference in basal synaptic transmission between WT and DISC1_tr_ slices (Fig.8A; *F* = 0.2, *P* = 0.7; repeated-measures anova; *n* = 26 WT and 20 DISC1_tr_ slices). Analysis of paired-pulse fEPSP amplitudes with respect to baseline showed a bell-shaped relationship with facilitation at all stimulation intervals and greatest facilitation at a stimulus interval of 30 ms (Fig.8B). Cross-genotype comparisons showed no difference in paired-pulse profiles, suggesting that presynaptic glutamate release is also not altered at TA-CA1 synapses by transgenic expression of DISC1_tr_ (fEPSP_2_/fEPSP_1_ *F* = 0.004, *P* = 0.9; repeated-measures anova; *n* = 12 WT and 9 DISC1_tr_ slices).

The same TBS protocol used to induce LTP in the SC pathway is also capable of producing LTP in the TA pathway. Figure8C plots fEPSP amplitude normalized to baseline for a 20-min period before the TBS conditioning stimulus and 60 min post-conditioning. In contrast to the SC pathway, TBS was able to induce significant LTP in WT slices but not in DISC1_tr_ slices (WT *t* = 2.8, *P* < 0.05; DISC1_tr_ *t* = 1.4, *P* = 0.2; one-sample, two-tailed Student’s *t* test). Comparison between genotypes revealed fEPSP amplitude was significantly smaller in DISC1_tr_ slices 1 h post-TBS. In WT slices, fEPSP amplitude was on average 1.34 ± 0.12 times greater baseline 1 h post-TBS, whereas in DISC1_tr_ slices fEPSP amplitude was 1.04 ± 0.03 times that of baseline at the same time-point. Statistical comparison showed that these differences were significant (*t* = 2.4, *P* < 0.05; unpaired, two-tailed Student’s *t* test; *n* = 13 WT and 11 DISC1_tr_ slices).

As in the SC pathway (Fig.7D), the area below baseline during the TBS LTP-induction protocol was measured to determine whether there were any differences during TBS that could account for the decreased LTP in DISC1_tr_ slices. In the TA pathway, area below baseline during the TBS protocol was very similar in WT and DISC1_tr_ slices (Fig.8D; *F* = 0.2, *P* = 0.6; repeated-measures anova; *n* = 13 WT and 11 DISC1_tr_ slices).

To ensure our stimulating electrodes placement was correctly producing selective activation of SC and TA pathways, and that there was no substantial cross-contamination of pathways, at the end of LTP experiments a subset of slices were treated with the group II mGluR agonist DCG-IV, which has been shown to suppress TA fEPSPs (Kew *et al*., 2001) but has no effect on SC fEPSPs (Kamiya *et al*., 1996). Whilst there was a small decrease in fEPSP amplitude in the SC pathway (WT 11.5 ± 2.5% inhibition; DISC1_tr_ 15.6 ± 2.6% inhibition), this was not significantly different between genotypes (*P* = 0.3; unpaired, two-tailed Student’s *t* test) and could be due to continued slow decline of responses following TBS (see mean post-TBS EPSP trajectory in top panel of Fig.7Cii). In the TA pathway, however, there was a clear and strong inhibitory effect of DCG-IV on fEPSP amplitude, confirming correct stimulating electrode placement. The degree of DCG-IV-mediated inhibition was not significantly different between WT and DISC1_tr_ slices (WT 78.6 ± 5.3% inhibition; DISC1_tr_ 67.4 ± 5.1% inhibition; *P* = 0.2; unpaired, two-tailed Student’s *t* test; *n* = 9 WT and 7 DISC1_tr_ slices), suggesting the presence of the transgene does not modify group II mGluR-mediated presynaptic depression.

## Discussion

The data presented here describe the first thorough study of intrinsic and synaptic properties of CA1-PNs in any DISC1-related mutant mouse model. The main findings are: (i) intrinsic neuronal properties are largely unaffected by transgenic expression of DISC1_tr_ with the exception of a propensity to exhibit higher rates of AP firing following the onset of a supratheshold depolarizing stimulus; (ii) in the SC pathway, whilst EPSP and IPSP summation, NMDA/AMPA ratios, basal synaptic transmission and short-term plasticity are not detectably altered in DISC1_tr_ mice, LTP is enhanced; and (iii) in the TA pathway, EPSP and IPSP summation, basal synaptic transmission and short-term plasticity are also unaltered in DISC1_tr_ mice, but LTP is abolished.

### Modifications to intrinsic properties

Altered intrinsic properties have been reported in mouse models designed to investigate psychiatric disease (Kehrer *et al*., 2007; Gisabella *et al*., 2009; Kvajo *et al*., 2011). Although most intrinsic properties were unaltered in DISC1_tr_ mice we did observe that CA1-PNs from DISC1_tr_ mice display an increased burstiness in the AP firing seen towards the start of a maintained depolarizing current injection (Fig.2). This effect was more pronounced with weaker, and arguably more physiologically relevant, current stimuli. For example, the mean instantaneous firing frequency of the first spike pair elicited with a 150-pA current injection was 59 ± 16 Hz in WT animals and 122 ± 23 Hz in DISC1_tr_ mice, a > 100% difference, whereas the equivalent frequency difference with a 300-pA stimulus was only ˜25%. This increase in initial firing rate, particularly with weaker current stimuli, is reminiscent of changes we have recently reported in CA1-PNs of two different amyloid beta-overproducing mouse lines widely used to model aspects of Alzheimer’s disease (Brown *et al*., 2011; Kerrigan *et al*., 2013).

The initial burst of higher frequency firing that occurs in CA1-PNs in response to depolarization is reported to involve certain voltage-gated conductances. These include the persistent Na^+^ current (*I*_NaP_) which contributes to the depolarizing phase of the ADP (Azouz *et al*., 1996; Yue *et al*., 2005) and the M-type K^+^ current (*I*_M_, mediated by K_V_7/KCNQ channels) which counteracts the depolarizing action of *I*_NaP_ and thereby controls the size of the ADP (Yue & Yaari, 2004, 2006). Enhancement of *I*_NaP_ or reduction in Kv7 channels could thus explain the phenotype of DISC1_tr_ mice. It would therefore be of interest to record *I*_NaP_ and *I*_M_ in CA1-PNs of DISC1_tr_ mice to determine whether changes in the ratio of these two currents could account for the increase in burst firing. Alternatively it would be of interest to investigate if drugs which either reduce *I*_NaP_, for example riluzole, or enhance Kv7 gating, for example retigabine, can normalize intrinsic excitability of CA1-PNs in DISC1_tr_ mice.

Whatever the underlying mechanisms involved in their generation, the intrinsic high-frequency burst firing properties of hippocampal pyramidal cells are thought to be important for multiple aspects of hippocampal function. For example, in presynaptic cells such firing is likely to promote LTP at downstream synapses, and in post-synaptic cells could also relate to induction of spike timing-dependent forms of plasticity.

### Enhanced LTP in the SC pathway

Our finding that LTP is increased in the SC pathway of DISC1_tr_ mice compared with age-matched littermate controls (Fig.7) contrasts with a study by Kvajo *et al*. (2008). They described a decreased level of post-tetanic potentiation but no difference in LTP. This previous study, however, employed both a different mouse model and a different LTP induction protocol (specifically a 100-Hz high-frequency tetanus), the latter being perhaps less physiologically pertinent than the TBS protocol we used.

In future it will be important to investigate the mechanism(s) underpinning the increased LTP in the SC pathway of DISC1_tr_ mice. There are many possible candidates, one of which is the increased burst firing of CA1-PNs we observed in DISC1_tr_ mice. Increased postsynaptic burstiness and dendritic backpropagation of these spikes following synaptic activation could lead to either greater or more prolonged membrane depolarization, in turn increasing NMDA receptor activation and Ca^2+^ influx during TBS, and thereby accounting for enhanced LTP. In support of this idea, several studies have demonstrated that pharmacological inhibition of K_V_7 channels, which increases postsynaptic burst firing, also facilitates the induction of LTP at SC synapses both *in vivo* (Song *et al*., 2009; Fontan-Lozano *et al*., 2011) and *in vitro* (Lampe *et al*., 1997; Petrovic *et al*., 2012).

Another mechanism with the potential to underpin the increased LTP in the SC pathway is changes to GABA-mediated synaptic inhibition. It has long been established that decreased signalling at GABAergic synapses facilitates LTP in area CA1 (Wigstrom & Gustafsson, 1985, 1986; Chapman *et al*., 1998). As yet we have not studied the intrinsic excitability and synaptic drive of CA1 GABAergic interneurons in DISC1_tr_ mice, although this is an important topic for future understanding of this model. Notably, however, the initial description of this mouse model demonstrated a significant reduction in parvalbumin staining in hippocampal area CA1 (and areas CA2 and CA3) (Shen *et al*., 2008), indicating a potential reduction in interneuron numbers. This therefore represents a good candidate to underpin the increased LTP described here. Furthermore, the fact that fEPSPs displayed a more ‘epileptic’ waveform following LTP induction in DISC1_tr_ mice (as quantified by measurements of fEPSP coastline; Fig.7Cii) could also be a consequence of decreased GABAergic input. It would therefore be of interest in future studies to examine whether the enhancement of SC LTP in DISC1_tr_ mice compared with WT is still apparent in the presence of GABA receptor antagonists.

Given that LTP is generally considered to be a cellular correlate of learning and memory (Bliss & Collingridge, 1993; Milner *et al*., 1998; Martin *et al*., 2000), an enhancement of SC LTP might at first suggest DISC1_tr_ mice should perform better in behavioural tasks involving this pathway than their WT littermates. Indeed, this may be the case and there are examples of mutant mice that display both enhanced SC LTP *in vitro* and enhanced performance in the Morris water maze task (a hippocampal-dependent spatial memory task) (Malleret *et al*., 2001; Chen *et al*., 2003). Contrary to this, however, there are also a number of studies in transgenic mouse models that show performance deficits in the Morris water maze accompanied by increases in SC LTP *in vitro* (Migaud *et al*., 1998; Uetani *et al*., 2000; Kaksonen *et al*., 2002; Kim *et al*., 2009; Earls *et al*., 2010). To our knowledge, experimental investigations of the performance of DISC1_tr_ mice in spatial memory tasks have not yet been published, and consequently it is only a matter of speculation as to what the functional consequences of the enhanced SC LTP in DISC1_tr_ mice may be.

### Deficits in TA LTP

In contrast to the enhanced LTP in the SC pathway, we observed a loss of LTP in the TA input to area CA1 in DISC1_tr_ mice (Fig.8). No genotype-related differences were observed in the field potential produced during the TBS protocol that could indicate changes in postsynaptic Ca^2+^ influx. TA synapses are formed on the deep dendrites of CA1 pyramidal cells and are reported to exhibit mechanistic differences from SC LTP. Some of these may relate to the more distal location of the synapses from the cell body. For example, in rats TBS-induced TA-LTP is not entirely blocked by NMDA receptor antagonism and has a component that is inhibited by a dihydropyridine, indicating a component of synaptic potentiation may arise from voltage-sensitive Ca^2+^ channel activation (Remondes & Schuman, 2003). The hyperpolarization-activated non-selective cation current *I*_h_ has also been shown to constrain TA LTP in an HCN1 knockout mouse (Nolan *et al*., 2004) and there is a substantial gradient of *I*_h_ channels along the length of the dendritic arbour of CA1-PNs. GABA_B_ receptor activation is also reported to be required for LTP in this pathway, unless GABA_A_ receptors are also blocked (Remondes & Schuman, 2003). Consequently, the differences between SC and TA LTP allow multiple places in which one could be specifically affected by a disease process. For example, a loss of L-type Ca^2+^ channel function would be predicted to inhibit TA LTP but not SC LTP and notably one class of L-type channel, α1C, is a known risk factor in psychiatric disease (Bhat *et al*. 2012). Also, the reported alteration in parvalbumin staining in hippocampal area CA1 of DISC1_tr_ mice (Shen *et al*., 2008) may indicate disturbed GABA_B_-mediated inhibition and thus a loss of TA LTP (Remondes & Schuman, 2003).

Whatever the mechanism underlying the deficit in TA LTP, this pathway is believed to play an important role in spatial information processing. An HCN1 knockout mouse displays enhanced performance in the Morris water maze accompanied by enhanced TA LTP but no change in SC LTP *in vitro* (Nolan *et al*., 2004). In rats with lesions of area CA3 (i.e. no SC input), CA1-PNs were still capable of developing distinct and stable place fields similar to those observed in control rats, suggesting that the direct pathway from the entorhinal cortex (TA pathway) provides spatial information required for proper place-cell formation and function, and that CA3 is not necessary for this process (Brun *et al*., 2002). However, these rats were impaired in the Morris water maze task, which points to an important role for intact CA3 input to CA1-PNs for spatial memory (Brun *et al*., 2002). In light of this, it would be interesting to examine whether there are alterations in the TA input in those transgenic mouse models that showed spatial memory deficits accompanied by enhanced SC LTP described above (Migaud *et al*., 1998; Uetani *et al*., 2000; Kaksonen *et al*., 2002; Kim *et al*., 2009; Earls *et al*., 2010) as both pathways play important roles in spatial information processing. Unfortunately, investigations usually focus on the SC pathway and the TA pathway is often overlooked, which is a surprising oversight because, as demonstrated here, both pathways can be easily studied in the same slice.

It is important to highlight that our studies of the intrinsic properties of CA1-PNs and studies of LTP were carried out in mice of different ages (3–4 months versus 8–9 months, respectively), so any ideas linking changes in intrinsic properties (such as enhanced burstiness of CA1-PNs) to altered synaptic plasticity must be treated very cautiously, and further studies are required to determine whether enhancement of SC-LTP and loss of TA-LTP is present at the younger age-point and/or whether increased burst firing is observed at the older age-point.

### Summary and future directions

The most striking finding of this study is the pathway-specific alterations in LTP in DISC1_tr_ mice, providing potential cellular mechanisms by which transgenic expression of DISC1_tr_ could disrupt normal hippocampal function. *In vivo* studies of appropriate hippocampal-dependent memory tasks are needed to determine whether these findings have functional consequences in behaving animals. Combining *in vivo* electrophysiological recordings with such behavioural tasks would provide further information about network activity and place cell function, both of which could be altered as a result of changes in synaptic plasticity in DISC1_tr_ mice. Together with thorough further investigations, the findings presented here could provide some insight into how truncation of DISC1 may affect CNS function in humans, for example features such as working memory in schizophrenia.

## Abbreviations

aCSF: artificial cerebrospinal fluid
ADP: after-spike depolarization potential
AHP: after-spike hyperpolarization potential
AMPA: α-amino-3-hydroxy-5-methyl-4-isoxazolepropionic acid
AP: action potential
CA1-PN: CA1 pyramidal neuron
CNS: central nervous system
DISC1: disrupted-in-schizophrenia 1
EPSC: excitatory postsynaptic current
EPSP: excitatory postsynaptic potential
fEPSP: field EPSP
GABA: γ-aminobutyric acid
IPSC: inhibitory postsynaptic current
IPSP: inhibitory postsynaptic potential
LTP: long-term potentiation
mAHP: medium AHP
NMDA: *N*-methyl-d-aspartate
*R*_i_: input resistance
RMP: resting membrane potential
sAHP: slow AHP
SC: Schaffer collateral commissural
TA: temporoammonic
TBS: theta burst stimulation
τ_M_: membrane time constant
WT: wild-type
